# Laser-Based Trace Gas Detection inside Hollow-Core Fibers: A Review

**DOI:** 10.3390/ma13183983

**Published:** 2020-09-09

**Authors:** Michal Nikodem

**Affiliations:** Department of Optics and Photonics, Wroclaw University of Science and Technology, Wybrzeze Wyspianskiego 27, 50-370 Wroclaw, Poland; michal.nikodem@pwr.edu.pl

**Keywords:** hollow-core fiber (HCF), negative curvature hollow-core fiber (NC HCF), photonic band-gap hollow-core fiber (PBG HCF), anti-resonant hollow-core fiber, Kagome hollow-core fiber, laser spectroscopy, gas sensing

## Abstract

Thanks to the guidance of an optical wave in air, hollow-core fibers may serve as sampling cells in an optical spectroscopic system. This paper reviews applications of hollow-core optical fibers to laser-based gas sensing. Three types of hollow-core fibers are discussed: Hollow capillary waveguides, photonic band-gap fibers, and negative curvature fibers. Their advantages and drawbacks when used for laser-based trace gas detection are analyzed. Various examples of experimental sensing systems demonstrated in the literature over the past 20 years are discussed.

## 1. Introduction

Laser spectroscopy has become a powerful tool in trace gas detection with a variety of applications, including environmental monitoring, breath analysis, industrial process monitoring, and leak detection. Laser spectroscopy in the near- and mid-infrared spectral regions offers a high selectivity, high sensitivity (typically down to parts-per-million (ppm) or parts-per-billion (ppb) levels), and short measurement time (usually less than 1 s). Additionally, optical systems operating in the infrared region may be equipped with thermo-electrically cooled (TEC) detectors and reliable and robust semiconductor sources (laser diodes, interband cascade lasers, or quantum cascade lasers), which enables out-of-lab operations with minimal maintenance. As a result, laser-based infrared gas sensors have been successfully used in various environments. 

**Figure 1:**
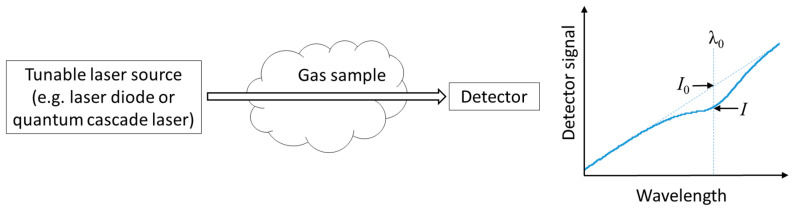
Schematic diagram of laser-based gas sensing.

Laser-based gas sensing primarily relies on measuring the absorption of light when its frequency/wavelength overlaps with the molecular resonance (as shown in Figure 1). This process is governed by the Beer–Lambert law:

*I* = *I*_0_·exp(−*σNL*), (1)

where *I*_0_ and *I* are the light intensities before and after the sample, respectively; *L* is the interaction length; *σ* is the absorption cross-section; and *N* is the number density (concentration). Based on above equation, one can deduce the molecular concentration simply by measuring the decay of the intensity after the light beam has passed through the sample (assuming that the interaction length and the absorption cross-section are known).

The Beer–Lambert law leads to simple conclusions on how to increase the sensitivity of the optical-based sensing system. For a given noise level, two possible ways are increasing the absorption cross-section and increasing the interaction length. The first one can be achieved by selecting the spectral region where stronger molecular transitions are located, e.g., for methane (CH_4_), absorption lines near 3.3 µm are ~50 times stronger than overtone transitions near 2.3 µm and ~100 times stronger than absorption lines close to the telecom range (near 1.65 µm). Therefore, performing detection in the mid-infrared spectral region will generally result in lower detection limits. Sensitivity enhancement may also be achieved by increasing the interaction length (*L*). This is often accomplished using multi-pass cells (MPCs). Some examples include White [1] and Herriott designs [2], astigmatic Herriot cells [3,4,5], multi-pass cells with spherical mirrors [6], and circular cells [7,8,9]. An important parameter of every MPC is the path length to volume ratio and it varies from a few centimeters per mL [5,7] to almost 0.25 m/mL for the most compact designs [6]. Unfortunately, in MPCs, a high compactness may lead to drifts and instabilities, as demonstrated in [10]. In this respect, optical fibers with a hollow-core seem to be an attractive alternative to MPCs. When a hollow-core fiber (HCF) is filled with a gas sample it may offer a long interaction length. At the same time, since an HCF core is typically on a micrometer scale (usually from a few to a few hundreds of micrometers), a very small sample volume is required. With fiber-based technology, one can also expect compactness (because the fiber can be coiled) and a good thermal stability and robustness.

The aim of this review is to collect and discuss examples of gas sensing systems that use HCFs as gas sampling cells. The paper comprises six sections. In Section 2, configurations of HCF-based gas sensing systems are discussed. Section 3, Section 4 and Section 5 review applications of three types of hollow-core fibers for gas sensing: Section 3 is focused on capillary tubes/waveguides with a reflective inner coating, Section 4 is devoted to hollow-core microstructure optical fibers in which the photonic band-gap (PBG) is responsible for light propagation, and Section 5 presents sensing setups that use Kagome and negative curvature hollow-core fibers (NC HCFs) which rely on an inhibited coupling (IC) guiding mechanism. Advantages, drawbacks, opportunities, and challenges associated with the use of each HCF type in laser-based gas sensing are discussed. Section 6 contains conclusions.

## 2. Configurations of HCF-Based Gas Sensing Systems

**Figure 2:**
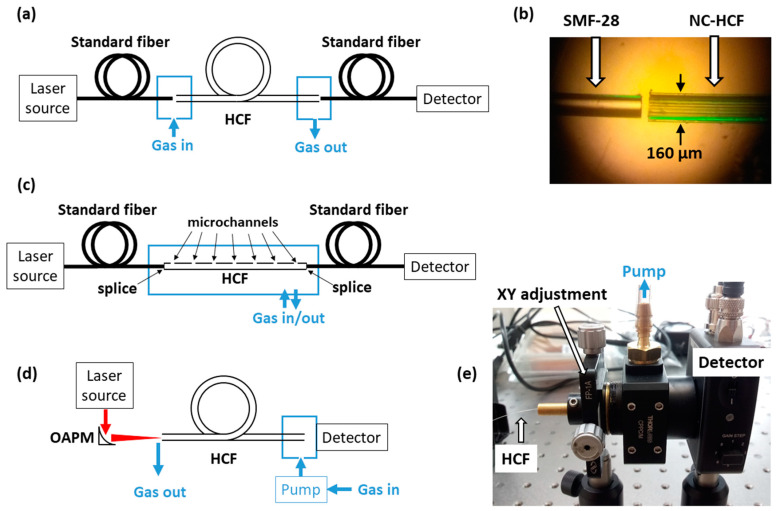
Potential configurations of hollow-core fiber (HCF)-based gas sensing systems: (**a**) HCF is butt-coupled with a standard single-mode fiber; (**b**) picture of a standard SMF-28 fiber coupled with negative curvature (NC) HCF; (**c**) HCF is spliced to a solid-core fiber from both sides, and gas enters/exits the fiber through microchannel drills along the HCF; (**d**) light is coupled into HCF using bulk optics, mirrors, or lenses (OAPM—off-axis parabolic mirror); (**e**) picture of air-tight housing which allows the HCF to be aligned with the photodetector and filled with a gas sample.

In order to perform gas sensing inside an HCF, it is necessary to couple the light in/out and, simultaneously, let gas enter and exit the fiber. One potential and common approach (shown in Figure 2a) is to butt-couple a standard single-mode fiber with HCF and leave a small (few micrometers to tens of micrometers) gap between them. This type of coupling is shown in Figure 2b. In this case, a coupling efficiency of 50 to 55% can typically be obtained [11]. An alternative solution is to splice a standard fiber with HCF and drill side microchannels to allow gas to access the hollow core (Figure 2c). Microchannels may be drilled using femtosecond laser pulses. Another approach is to use side coupling, which was recently demonstrated in [12]: A standard single-mode fiber and HCF were twisted together and pulled in opposite directions after being heated using a hydrogen flame. The advantage of this solution is that the hollow core remains open and undisturbed.

Fiber-to-fiber coupling can be used in the near-infrared range, at 1.55 or 2 µm. When HCFs are used in the mid-infrared region, free space coupling is necessary, using, e.g., mirrors (Figure 2d) or lenses. Some kind of air-tight housing which will allow flowing gas through the fiber must also be designed. An example is shown in Figure 2e.

## 3. Gas Sensing Using Capillary Fibers/Waveguides

**Figure 3:**
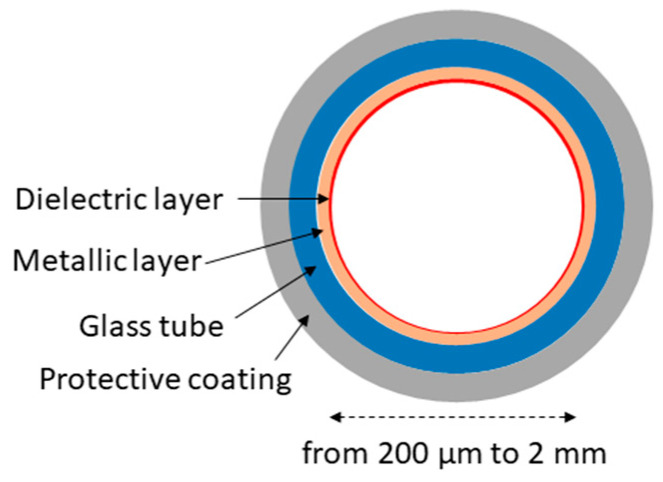
Schematic diagram of the construction of a typical glass hollow waveguide. Metallic and dielectric layers are usually made of silver (Ag) and silver iodine (AgI), respectively.

Early work on hollow capillary fibers/waveguides was conducted in the 1960s [13]. Their main advantage is that good transmission in the mid-infrared can be obtained relatively easily, i.e., by choosing an appropriate inner coating of the fiber. Hollow waveguides made of glass [14], plastic [15], and even metal [16] have been demonstrated, with their inner surface coated with a metallic layer (usually silver) and dielectric film (for enhanced reflectivity). Delivering high power beams from CO_2_ lasers (which emit near 10.6 µm) was the main motivation for the progress in this field [14,17]. Figure 3 shows a schematic diagram of the construction of a typical glass hollow waveguide.

**Figure 4:**
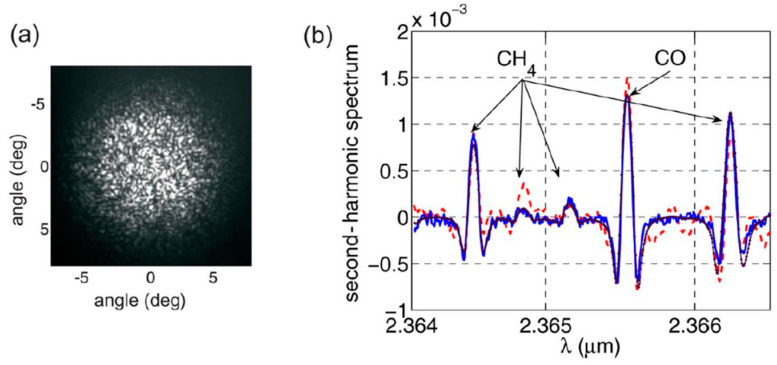
(**a**) A far field of the hollow waveguide/fiber used in [20]. Speckle pattern indicates multimode propagation inside HCF. It led to the presence of optical fringes in the recorded spectra, which could be washed out by applying vibration to the fiber; (**b**) wavelength modulation spectroscopy (WMS) spectra (second harmonic signal) measured with and without vibration of the fiber (blue/solid and red/dashed curves, respectively). Also shown is the simulated/theoretical spectrum (black/dotted). Reprinted with permission from [20], © The Optical Society.

Some of the earliest work on gas sensing inside flexible hollow capillary fibers was demonstrated in [18] and later in [19]. In both cases, absorption spectroscopy was performed in the near- and mid-infrared spectral regions. Multipath propagation in the waveguide with a relatively large diameter (1 mm [18]) was found to produce background noise which could be suppressed when a capillary with a smaller size was used [19]. A similar noise issue was found by Chen et al. in [20]. In their setup, a 2.3 µm vertical-cavity surface-emitting laser (VCSEL) enabled targeting the molecular transition of carbon monoxide. A 3-m-long fiber with an inner diameter of 750 µm was used (1.2 mL volume). The detector housing was filled with a reference gas (methane) that served as a wavelength reference. The multimode propagation inside the hollow fiber (indicated by the speckle pattern in the far field visible in Figure 4a) produced background signals in the measured spectra which could only be removed by applying vibrations to the fiber end and integrating the recorded signal. Sample absorption spectra recorded using wavelength modulation spectroscopy (WMS) are shown in Figure 4b. The absorption features of carbon monoxide (inside HCF) and methane (inside photodetector housing) are visible, with and without vibration of the fiber. Despite issues related to multimode propagation, the presented setup was capable of resolving absorbance of less than 10^−4^, which was comparable to the performance obtained using conventional gas cells.

**Figure 5:**
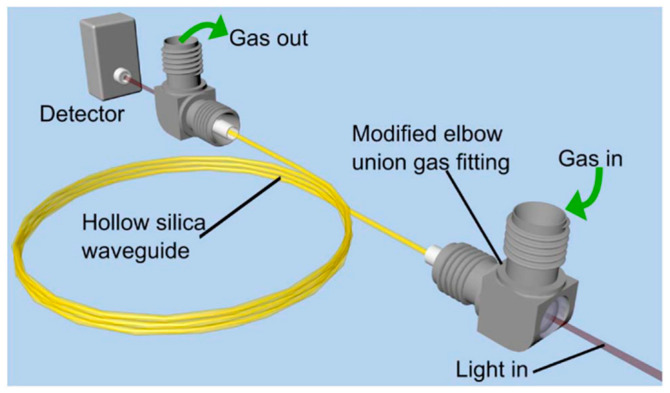
Schematic diagram of the gas cell based on a hollow capillary fiber/waveguide with elbow fittings used to couple gas and light in and out of the fiber. Reprinted with permission from [24], © The Optical Society.

**Figure 6:**
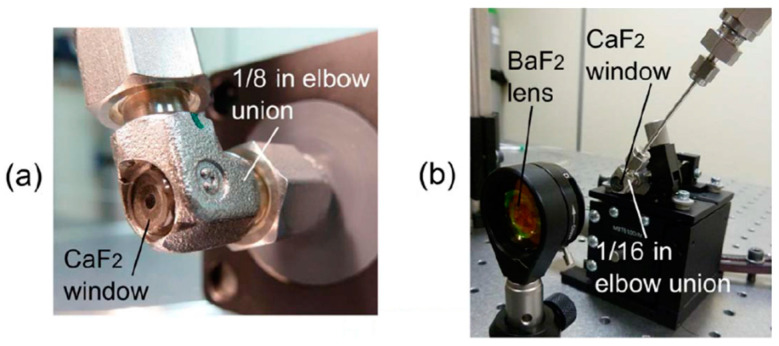
Photographs of the gas fitting ports used in [24]. Reprinted with permission from [24], © The Optical Society.

The issue of optical fringes was later addressed by the same group by employing Zeeman modulation spectroscopy [21]. In this technique (which is essentially very similar to Faraday rotation spectroscopy [22]), a sample is located inside the magnetic coil and the presence of the magnetic field causes the molecular transitions to split. As successfully demonstrated in [21], this allows the gas sample to be modulated (when a modulated magnetic field is used) and enables one to distinguish between the modulated spectral signature of the molecule and unmodulated background. Unfortunately, Zeeman spectroscopy can only be used for paramagnetic species (e.g., O_2_, NO, or NO_2_), so its potential applications are limited. A similar hollow fiber was also used in [23], where a broad transmission bandwidth of the waveguide enabled methane transitions near 3.3 and 7.7 µm to be simultaneously recorded. More recently, laser-based gas sensing inside hollow waveguides has been demonstrated using quantum cascade lasers (QCLs) operating in the mid-infrared spectral region. In [24], pulsed QCL operating at 7.82 µm was used to detect methane inside 5- and 10-m-long hollow waveguides. A short response time (less than 1 s for a 5-m-long fiber with a 1000 µm inner diameter) and a very decent limit of detection were obtained. It is also worth mentioning the coupling of HCF used in this work. As shown in Figure 5, both ends of the fiber were placed in elbow gas fittings from Swagelok. They were modified, enabling light coupling in and out of the fiber, as presented in Figure 6.

In [25] and [26], broadly tunable external cavity QCLs were coupled in a 3-m-long hollow fiber. A similar approach was also used in [27], but the length of the hollow waveguide was only 2.5 cm. In these three examples, widely tunable lasers were used for broadband spectral analysis. It is particularly worth noting the simultaneous multi-species measurement that was demonstrated in [26], which takes full advantage of the broad transmission bandwidth offered by the capillary fiber with an inner surface coated with silver. In [28], a photothermal interferometry (PTI) technique that benefits from this spectral property of hollow waveguides was shown. In this work, the two waves, the first at 4.46 µm and the second at 1.55 µm, were simultaneously coupled in the 25-cm-long hollow fiber. The mid-infrared beam was modulated and used as a pump source. Due to the photothermal effect, the absorption of modulated light produced changes of the effective refractive index inside the fiber, which were measured as phase variations of the probe (near-infrared) beam. A detection limit of less than 1 parts-per-million (ppm) at a 1 s integration time was obtained using nitrous oxide as a target molecule and a pump power of only 6 mW. Other interesting demonstrations of laser spectroscopy inside a hollow fiber/waveguide are shown in [29] and [30]. In [29], the real-time measurement of isotopologue ratios is presented. This type of measurement is very challenging since it requires a high sensitivity and high precision at the same time. In [30], a setup for the mid-infrared sensing of methane is demonstrated, with a 3.3 µm light emitting diode (LED) as a source. This combination provides a perspective for simple, low-cost, and fiber-based mid-infrared gas sensors.

To summarize this section, broad transmission is the biggest advantage of hollow capillary waveguides when application to gas sensing is considered. It not only allows one to target strong, fundamental transitions located in the mid-infrared range, but also enables one to measure broad absorption features or detect multiple species simultaneously. Over the last 20 years, several examples of laser spectrometers based on flexible hollow capillary waveguides have been reported. Setups that operate in the near- and mid-infrared spectral regions and use various spectroscopic techniques have been demonstrated.

## 4. Gas Sensing Inside Photonic Bad-Gap Hollow-Core Fibers

**Figure 7:**
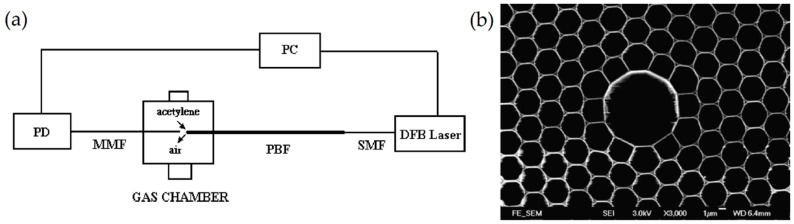
(**a**) Schematic diagram of the experimental setup used in [32]; (**b**) microscopic picture of the HCF used in this work (the fiber was obtained from Blazephotonic, UK). Reprinted from [32], with permission from Elsevier.

Light guiding inside a hollow-core photonic crystal fiber (PCF) was demonstrated for the first time in 1999 [31]. A few years later, PBG HCFs attracted the attention of researchers working on optical-based gas sensing. The first basic experiments were reported in 2014 by Hoo et al. in [32]. Figure 7 shows the experimental setup and cross-section of the PBG-HCF used in this work. The light from a distributed feed-back (DFB) laser diode was coupled in the HCF by splicing it to a standard single-mode fiber (SMF). The other end of the HCF was placed inside a tight gas chamber. A multi-mode fiber (MMF) was used to collect the light from the HCF and deliver it to the photodetector (PD). A small gap (approximately 50 µm) between the HCF and MMF was left to enable gas diffusion into the HCF. The DFB laser wavelength was periodically scanned across the absorption line of acetylene (C_2_H_2_). When pure acetylene was blown into the gas chamber, a minimum of the transmittance was recorded with the photodetector.

A few months after the first demonstration, Ritari et al. presented optical spectroscopy inside 1- and 10-m-long PBG HCFs [33]. The absorption spectra of acetylene (near 1.52 µm), hydrogen cyanide (near 1.535 µm), and methane (near 1.33 µm) were recorded using light emitting diodes (LEDs) as sources and an optical spectrum analyzer as a detector. Between 2007 and 2009, Cubillas et al. demonstrated the first laser-based high-resolution spectroscopy inside PBG HCF [34,35,36]. The 5.1-m-long fiber was made by the Optoelectronics Research Centre in Southampton (UK) and had a core diameter of 12 µm. A 1645 nm light from a tunable laser was used to measure the absorption lines of methane. The laser light was directly coupled in the HCF from a standard single-mode fiber that was cleaved and placed very close to the HCF end. A small gap that was left between the two fibers allowed gas to enter and exit the HCF. The other end of the HCF was spliced to a standard single-mode fiber and the signal after the sample was analyzed with an optical power meter. After the fiber was filled with the mixture of methane and nitrogen, multiple absorption lines of methane could be measured. These early works exposed two major issues, which will be tackled and addressed in almost all further papers on gas sensing inside PBG HCFs: Long gas filling times and optical fringes.

The first issue is the gas diffusion time inside the fiber. Recently, this subject was analyzed numerically and experimentally by Masum et al. [37]. In this work, the gas flow inside the hollow-core fiber was modeled using a numerical model based on the Navier–Stokes and diffusion equations. The model was validated with experimental analysis. According to these studies, the gas filling time is affected by various experimental parameters, such as the HCF diameter and length, gas parameters (e.g., viscosity or density), and pressure difference between the inlet and outlet of the HCF. The authors demonstrated the possibility of reducing the gas filling time for a 1.1-m-long PBG HCF with a core dimeter of 10 µm to less than 30 s. However, this required using a very high pressure difference (4 atm). In practice, the filling times reported in many other papers are longer, e.g., in [35], it took 7 min to fill a 5.1-m-long fiber with a gas sample. Some other results reported in the literature are summarized in Table 1, where filling times vary from a few minutes up to even more than an hour.

**Figure 8:**
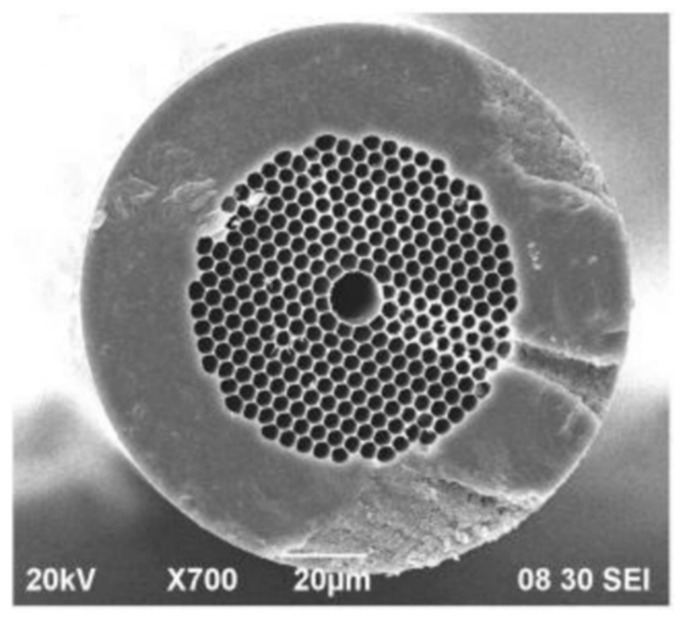
Scanning Electron Microscope image of a hollow-core fiber with a side microchannel. Reprinted from [45].

Two main approaches for tackling the challenge of long filling times can be found in the literature. Parry et al. proposed dividing an HCF into sections, so that each section is filled independently [43]. A similar idea was also demonstrated in [44]. However, this solution eliminates some of the benefits of using HCFs instead of multi-pass cells (e.g., connections between segments of the fiber will be prone to thermal drifts). The second way to improve the response time of the PBG HCF-based gas sensor is to drill side microchannels which allow gas to enter the hollow core from the sides of the fiber. Figure 8 shows a cross-section of the fiber used in [45] with a visible microchannel. Other examples were presented in [46] and [47]. In the first paper, a 7-cm-long HCF with seven vertical microchannels was demonstrated. Microchannels were drilled using 120 fs pulses and had a diameter of 10 µm. The laser wavelength was stabilized at the center of the methane transition near 1665.5 nm. The light from a laser was sent through the HCF that was spliced to the SMF on both ends and placed inside a gas chamber. The optical power after passing the HCF was measured with the photodetector: The higher the methane concentration inside the HCF, the lower the voltage at the output of the detector. Seven side holes helped to reduce the gas filling time from almost one minute to only 3 s, without a significant increase of transmission losses. In [47], 242 microchannels were drilled along a 2.3-m-long HCF, resulting in a response time of only approximately 40 s.

**Figure 9:**
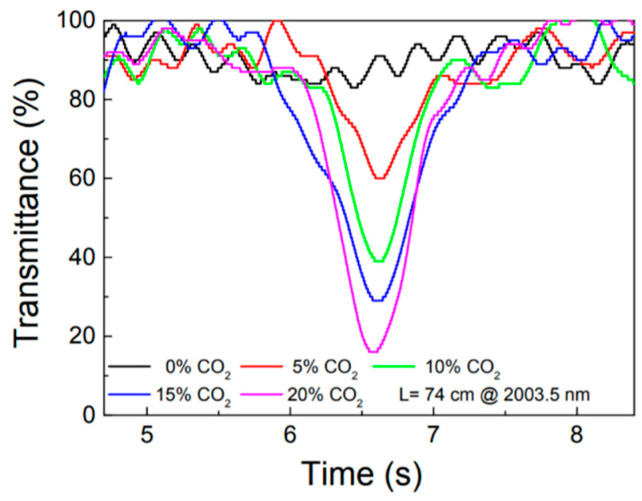
Photodetector signals recorded as the laser diode were tuned across the carbon dioxide transition. Spectra were measured for different CO_2_ concentrations. In each spectrum, optical fringes are strong and clearly visible. Reprinted from [39].

The second issue which can be noticed in the early work shown in [34] is the presence of interference fringes in the recorded spectra. The authors said that these background signals are due to reflections from the fiber facet. However, later papers on laser spectroscopy inside PBG HCFs suggest that the origin of these fringes is the propagation of higher-order spatial modes which interfere at the output. Similar background signals can be found in many other papers (e.g., Figure 9 shows spectra presented in [39]), and various ways to suppress them have been proposed. For example, in [43], the HCF was thermally cycled so that the fringe pattern could be washed out through averaging. Some improvement was obtained, but the demonstrated heating cycle was very long (a few minutes).

**Figure 10:**
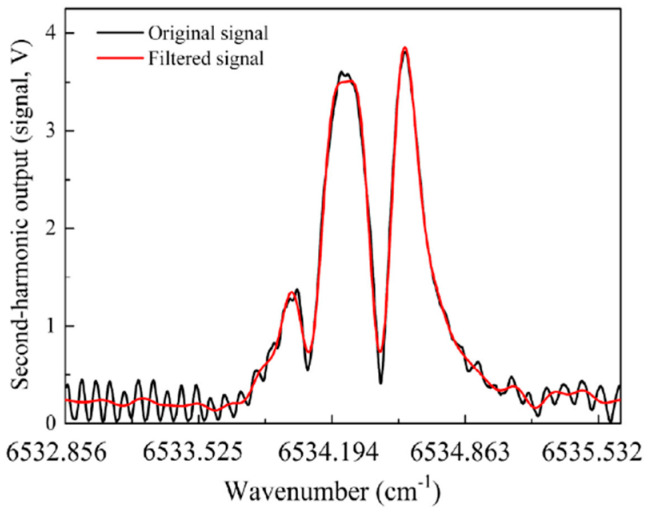
WMS signal (second harmonic) presented in [48]. Optical fringes (visible in the background of the original signal) could be removed in post-processing. Reprinted with permission from [48], © The Optical Society.

In [42], two lenses were used to optimize the light coupling in the HCF. A reduction of mode interference was reported, but the complexity of the setup increased. A few different approaches to fringe reduction were also proposed by Yang et al. In [48], not only was the spacing between the standard single-mode fiber and the HCF adjusted and optimized, but an additionally measured signal was post-processed to filter-out the fringe pattern. An example of original and filtered signals is shown in Figure 10. Unfortunately, this approach can only be applied when full spectral scans are recorded (which is often not the case when the WMS technique is used) and when the fringe pattern is significantly more dense than molecular spectral features.

In [47], the HCF was spliced from both sides and the gas sample entered the fiber through side holes. In this case, a large mode-area (LMA) fiber (placed between the standard fiber and the HCF) served as a filter for higher-order spatial modes. On the other side, the HCF was directly spliced to a standard fiber, but the process of splicing was carefully optimized: By changing the current of the arc fusion splicer collapse of the cladding, air holes could be controlled and adjusted so that higher-order modes were attenuated and mode interference was suppressed.

Some researchers decided to avoid the issue of optical fringes by using spectroscopic techniques which, to some degree, suppress these unwanted background signals, e.g., in [45], photothermal interferometery was used. This technique (already described in the previous section) is essentially background-free, i.e., if there are no molecules, there will be no photothermal signal (from gas or fringes). Therefore, when this method is used, the contribution from mode interference inside the HCF is expected to be much less troublesome compared to techniques in which absorption is measured directly. Another example where an appropriate detection method can be used to suppress optical fringes was demonstrated by Jaworski [40], who used chirped laser dispersion spectroscopy (CLaDS) to measure the molecular transition of carbon dioxide near 1572 nm. Fundamentally, CLaDS is a technique where molecular information is encoded into the phase difference between multiple waves that are sent through the sample. If the spacing between those waves is chosen appropriately (so that it matches the period of the fringes), an unwanted interference pattern can be suppressed. In [40], this approach was demonstrated for two different lengths of the HCF (1 and 20 m).

PBG HCF-based gas sensing has mainly been performed in the spectral region between 1530 and 1700 nm (HC-1550-02 fiber from NKT Photonics, shown in Figure 8, is by far the most popular choice, and has been used in multiple papers presenting HCF-based gas sensing [40,41,42,43,45,46,47,48,49,50]). However, as mentioned in the introduction, the mid-infrared spectral region offers stronger molecular transitions. Therefore, performing sensing and spectroscopy at a longer wavelength is highly desired. In 2005, Shephard et al. reported the first silica-based PBG hollow-core fiber with guidance beyond 3 µm [51]. A few years later, the same group demonstrated mid-infrared spectroscopy inside an 80-cm-long PBG HCF, using an optical parametric oscillator (OPO) as a source and a Fourier transform spectrometer (FTIR) as a detector. The potentially achievable detection limit was roughly estimated as 50 ppm of methane [52]. Continuation of this work can be found, e.g., in [53], where the spectra of acetylene (near 3.05 µm) and ethane (near 3.35 µm) were recorded using a supercontinuum source and optical spectrum analyzer. Good agreement between the measurement and theoretical predictions (based on HITRAN and Pacific Northwest National Laboratory databases) was achieved. However, although both papers ([52] and [53]) present mid-infrared optical spectroscopy inside PBG HCFs, they should be primarily considered as proof-of-concept demonstrations. In both cases, precise characterization of the system performance (e.g., in terms of the detection limit or linearity) was impossible because only highly absorbing samples were measured and spectra were recorded with a low optical resolution.

Table 2 summarizes the advantages and drawbacks of using PBG HCFs as gas cells in trace gas sensing. PBG HCFs have been commercially available for many years and exhibit very low losses, especially in the near-infrared spectral region. They are flexible and robust and can be easily spliced to standard single-mode fibers using ordinary fusion splicers. Some approaches for suppressing mode interference have been proposed. Unfortunately, the filling times are very long (a few minutes and more), even when relatively short (less than 1 m) PBG HCFs are used. The response time of HCF-based sensors can be improved by drilling multiple microchannels along the fiber, but it is hard to imagine that this approach can be successfully used in out-of-lab environments, where, e.g., the sensor is exposed to dust.

The slow response time of the system is not an issue when a gas-filled HCF is used as a permanent gas cell, which can be used, e.g., as frequency reference in optical metrology. This requires an HCF to be filled with gas and sealed from both sides (e.g., through splicing to a standard fiber). In this scenario, the most important drawbacks of PBG HCFs are not present. The slow response time is not an issue anymore because the fiber only needs to be filled once. Additionally, because a sample with a high concentration can be used, the contribution from optical fringes is not as troublesome as in trace-gas detection. Some examples of PBG HCF-based reference gas cells can be found in [54,55,56,57,58,59].

## 5. Trace-Gas Detection Using Hollow-Core Fibers with Inhibited Coupling Guidance

**Figure 11:**
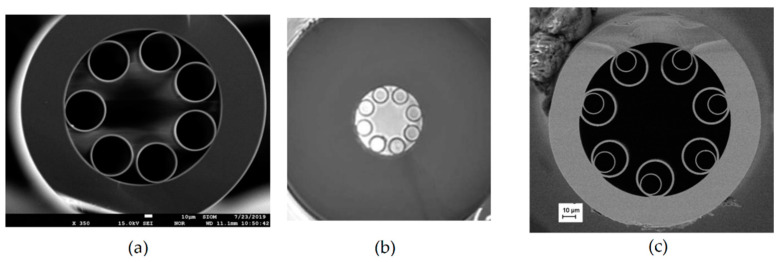
SEM images of examples of NC HCFs: (**a**,**b**) revolver/tubular HCFs; (**c**) revolver/tubular HCF with double nested capillaries. (**a**) Reprinted from [65]; (**b**) reprinted with permission from [66], © The Optical Society; (**c**) reprinted with permission from [67], © The Optical Society.

The Kagome fiber was presented for the first time in 2002 [60]. In its very first demonstration, the Kagome HCF was already filled with gas (hydrogen in that case). However, the aim of this study was not to perform optical spectroscopy, but to analyze nonlinear effects inside the gas-filled HCF. The Kagome HCF guides light due to the coupling between core and cladding modes (so-called inhibited guidance mechanism), rather than due to photonic bandgaps [61]. Many authors also agree that antiresonance plays an important role in light guidance inside NC HCFs [62]. Further studies in this field led to the discovery that transmission losses primarily depend on the core wall shape [63] and demonstration of the simplified, tubular structure of the fiber [64]. SEM images of cross-sections of three different NC HCFs are shown in Figure 11.

Negative curvature HCFs have few advantages compared to PBG HCFs, including broader transmission (divided into several bands) and lower overlap between light and glass material. The latter makes it easier to obtain good transmission in the mid-infrared region, even when silica is used as a host glass. Both features can be particularly beneficial when NC HCFs are used in optical spectroscopy, especially in photothermal interferometry, where two different wavelengths have to be coupled in the fiber, e.g., one in the mid-infrared region (pump light) and the other in the near-infrared region (probe light). Additionally, the core size in Kagome or tubular/revolver-type HCFs is typically larger than in PBG HCFs, which should result in shorter gas filling times (recent studies suggest that this is true, even when fibers with similar core sizes are compared [68]). This property of NC HCFs was confirmed in a 2016 conference paper by Challener et al. [69]. A 1.5-m-long fiber from the University of Bath (UK) was used to measure the absorption of methane using tunable diode absorption spectroscopy (TDLAS). Although not many details about the system were provided in this paper, the authors have shown that when a small pressure difference between the two fiber ends is applied, the gas diffusion time through the fiber is reduced to only a few seconds. A more detailed analysis was performed in [70]. A light from a differential frequency generation (DFG) source operating near 3.33 µm was coupled in a 1.3-m-long Kagome HCF from GLO Photonics (France). The other end of the fiber was placed in air-tight housing, which enabled the HCF to be filled with gas (air, nitrogen, or methane/nitrogen mixture). A CaF_2_ lens was used to focus the light onto a mercury-cadmium-telluride (MCT) detector. Using a WMS technique, a minimum detection limit of 4.11 ppm was obtained. This result was limited by optical fringes and when fringes were removed (through post-processing), the detection limit of ~0.5 ppm was achieved. More importantly, the sensor response time was less than 10 s. The same fiber was also used in [71], but this time, it was combined with the CLaDS technique. In this case, a detection limit of less than 1 ppm was obtained for the integration time of 1 s (and 65 ppb when averaged for 100 s).

**Figure 12:**
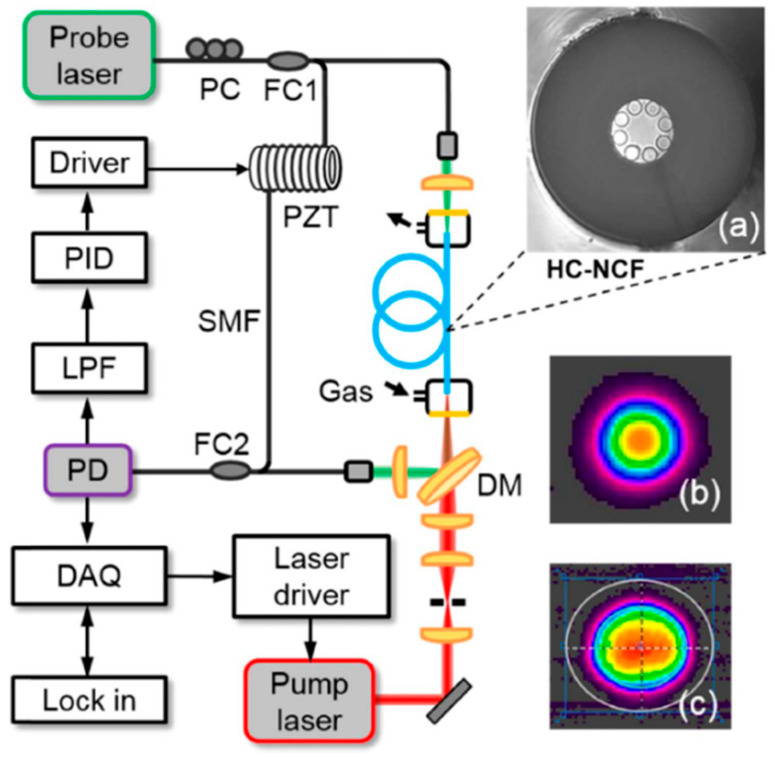
A schematic diagram of the photothermal interferometry (PTI) setup from [66]. A distributed feed-back (DFB) laser diode at 2.3 µm was used as a pump laser. The interferometric measurement was performed at 1.55 µm (pump laser). PD—photodetector, LPF—low-pass filter, and DAQ—data acquisition card. (**a**) shows an SEM image of the fiber, and (**b**,**c**) show the beam profile of the pump beam after and before passing through the hollow-core fiber (HC-NCF), respectively. Reprinted with permission from [66], © The Optical Society.

In [11], laser spectroscopy inside a tubular (revolver) NC HCF was presented. A 1.35-m-long fiber with a core dimeter of 70 µm was made in the Institute of Electronic Materials Technology (Poland). The fiber had two transmission bands in the near-infrared region, from 1350 to 1600 nm and from 1790 to 2220 nm. In a gas sensing experiment, a discrete mode (DM) laser diode operating near 2004 nm was used. The standard single-mode fiber at the output of the laser diode was butt-coupled to the NC HCF (Figure 2b). The coupling efficiency was approximately 50%. The other end of the HCF was placed inside the custom-made housing, enabling the fiber to be filled with a gas sample. A WMS technique was used for concentration monitoring. A minimum detection limit of 5 ppm (3 s averaging) and a response time of 5 s were obtained. It has also been demonstrated that significant suppression of the optical fringes can be achieved when coupling between the input fiber and HCF is carefully adjusted. In [72], Yao et al. presented a setup for carbon monoxide sensing at 2.3 µm using direct laser absorption and WMS. A gas filling time of 5 s was measured and a detection limit of only 0.4 ppm was obtained (30 s averaging). The same fiber was also used in [66], where carbon monoxide was detected with PTI. The system used in this paper is shown in Figure 12. A 1.55 µm light was used as a probe to measure the photothermal signal employing an interferometer formed between two fiber couplers (FC1 and FC2). A hollow-core fiber was placed in one arm of the interferometer. A dichroic mirror (DM) was used to couple the mid-infrared light in the HCF. A piece of standard fiber was coiled around a piezo transducer (PZT) and was used to make sure that the interferometer operated at the quadrature point.

**Figure 13:**
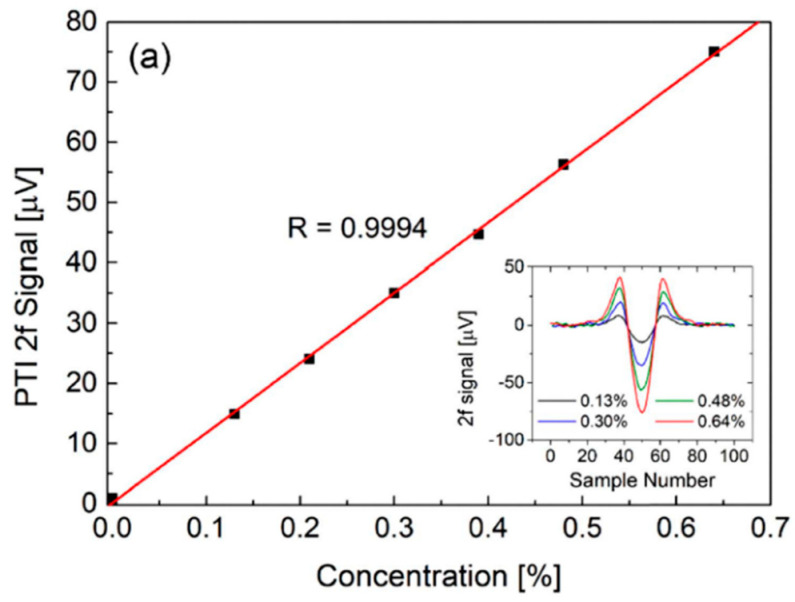
Linear response of the PTI sensor shown in [66] to the CO concentration. Inset shows the PTI spectra measured at different CO concentrations. Reprinted with permission from [66], © The Optical Society.

Figure 13 demonstrates the photothermal signal as a function of the carbon monoxide concentration and the inset shows the PTI spectra recorded for four different samples. A linear response and noise equivalent concentration of less than 100 ppm were reported.

In [73], the same group presented another system, operating at 3.6 µm. The fiber was 1.2-m-long, had a core diameter of 65 µm, and had a transmission band from 1.6 µm to the mid-infrared region. An interband cascade laser (ICL) was used to detect nitrous oxide (N_2_O). A detection limit comparable to standard spectroscopic systems was obtained. The authors also pointed out that, in order to reduce optical fringes, light coupling conditions must be optimized.

Photothermal spectroscopy inside an NC HCF was also recently presented in [74] and [75]. The experiments in [74] were performed near 1.53 µm. Two fiber-based cells were used, made with 4.67- and 0.74-m-long NC HCFs. A response time of 44 s was measured for the shorter cell, but in this case, the gas did not enter through the end of the HCF, but through microchannels drilled from its side. The setup in [75] used a shorter piece of the NC HCF (a few cm). It was placed between the SMF to form a Fabry-Perot interferometer. Again, the sensing was performed near 1.53 µm. Not only was the long term performance analyzed, but also the impact of the modulation frequency in PTI on the signal amplitude was investigated.

**Figure 14:**
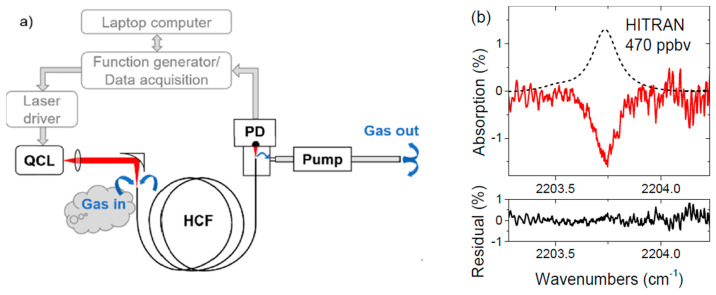
(**a**) A schematic diagram of the setup used in [67] for the laser spectroscopy of nitrous oxide. The hollow-core fiber used in this work has already been shown in Figure 11c; (**b**) direct laser absorption spectrum (solid line) and model (based on HITRAN database; dashed line) for the trace concentration of N_2_O. Fitting residual is shown below. Reprinted with permission from [67], © The Optical Society.

As mentioned above, NC HCFs have the ability to transmit light in the spectral region, where losses of the glass material are relatively high. This property was demonstrated in [67], where a silica-based NC HCF was used to detect nitrous oxide near 4.53 µm. The fiber used in this study (made in the Institute of Electronic Materials Technology, Poland) was optimized for light transmission near 4 µm and had exceptionally low bending losses [76]. A schematic diagram of the sensor used in [67] is shown in Figure 14a. A 3.2-m-long fiber was used for gas sensing. A light from a distributed feedback QCL was coupled in the HCF using an off-axis parabolic mirror. The other end of the fiber was positioned in front of the MCT detector (as shown in Figure 2e). Tight housing connected with a pump was built to enable gas flow through the fiber. The sample absorption spectrum measured when the laser wavelength was scanned across the nitrous oxide transition near 2203.75 cm^−1^ is presented in Figure 14b. When the WMS technique was implemented, the detection limit of a few parts-per-billion was obtained and the response time was 23 s.

The NC HCFs described above were made of fused silica and enabled molecular transitions up to 4.5 µm to be achieved [67]. Transmission at even longer wavelengths has been obtained using other glass types. NC HCFs made of various materials have been demonstrated, including borosilicate [77], tellurite [78], and chalcogenide [79,80,81] glass. In [82], a 1.15-m-long borosilicate NC HCF with a core dimeter of more than 100 µm was used for gas sensing near 5.26 µm. A QCL was used as a source to detect nitric oxide (NO). A gas filling time of approximately 9 s was obtained, which is consistent with other published results.

Table 3 summarizes examples of using NC HCFs for trace gas sensing. With only one exception, all described experiments were performed for wavelengths beyond 2 µm. Multiple groups presented optical spectroscopy of fundamental ro-vibrational transitions (i.e., beyond 3 µm) with detection limits at ppm or ppb levels for various molecules, including methane and nitrous oxide (both potent greenhouse gases) and nitric oxide (a hazardous pollutant). More importantly, the reported gas diffusion times were below 10 s for fiber lengths of approximately 1 m. For the 3.2-m-long HCF presented in [67], a 23 s time was obtained, which is still acceptable in many trace gas detection applications.

It should also be mentioned that there are several papers which show Kagome HCFs used as permanent gas cells [83,84,85]. These experiments were primarily performed in the near-infrared region, at around 1.55 µm (with the HCF filled with acetylene) or near 2.05 µm (where absorption lines of carbon dioxide were used as the frequency reference).

## 6. Conclusions

This paper summarizes the examples of using hollow-core fibers for trace gas detection in the near- and mid-infrared spectral regions. Setups that use three types of fibers (hollow capillary waveguides with an inner reflective coating, photonics band-gap fibers, and negative curvature fibers) have been described. With all HCFs, low transmission losses (below 1 dB/m) can be obtained. This is acceptable in many sensing applications in which the required interaction length is up to a few meters.

The biggest advantage of hollow waveguides is broadband transmission, which makes them particularly useful in the mid-infrared region, where the strongest molecular transitions are located. Moreover, those waveguides have a relatively large core diameter, which becomes beneficial when they need to be filled with a gas sample. This is different with photonic band-gap HCFs. In their case, the reported filling times vary from a few to a few tens of minutes, even for short (~1 m) pieces of fiber. Additionally, strong optical fringes are typically present when PBG HCFs are used, which directly impacts the measurement sensitivity. However, because PBG HCFs are commercially available and can be relatively easily spliced to standard single-mode silica-based fibers, they can be conveniently transformed into all-fiber-based permanent gas cells and used, e.g., as reference frequency standards. In this application, the fiber only has to be filled once, which eliminates the biggest drawback of PBG fibers.

In the last two years, several papers have reported trace gas sensing inside negative curvature HCFs. These fibers have broader transmission than PBG HCFs, produce relatively small optical fringes, and have reasonably short filling times. If necessary, they can also be spliced to standard solid-core fibers. Rapid progress has been observed over the last few years in terms of developing NC HCFs with small bending losses and good transmission also in the mid-infrared region. These features make them very promising components for compact and robust trace-gas detection systems, particularly in applications such as environmental monitoring, where an instantaneous response time is not always critical.

Sensitivity is an important parameter of gas sensing systems and the detection limit was determined in almost every paper presenting gas sensing inside an HCF. Unfortunately, the methodology was not consistent among papers, which often makes it difficult to compare reported results and accurately evaluate the performance of HCF-based sensors. However, in some papers (e.g., [20] or [71]), a comparison with standard, free-space systems was provided. The results obtained in these papers suggest that when optical fringes are suppressed (e.g., using proper alignment or by applying vibrations to the fiber [20]), the detection limits obtained using an HCF are similar to those achieved with free-space sensing. Moreover, HCFs become particularly useful when photothermal spectroscopy is used. In this case, due to tight light focusing and a long interaction length, the sensitivity can be much higher than in free-space detection [45].

Currently, the response time of the HCF-based sensor seems to be the biggest challenge. Even though hollow capillary waveguides and NC HCFs provide much shorter diffusion times compared to PBG HCFs, it may be difficult to obtain an instantaneous response when an HCF that is a few meters long is used. Another aspect related to the response time that has not been discussed in detail so far is the sampling issue. Some molecules can bind to the inner wall of the HCF, which may eventually lead to measurement inaccuracies. This issue was recently analyzed in [37]. However, it would be interesting to see systematic experimental measurements with some ‘sticky’ molecules, e.g., ammonia.

Apart from the many experimental results that have been published over the last 15 years, some authors have also reported theoretical analyses devoted to gas sensing inside hollow-core fibers. In [37], a numerical model of gas flow through HCF was described. In [86], the single-mode performance of NC HCFs was analyzed numerically. In [75], a simple analytical model was developed to describe the dynamics of the signal in photothermal spectroscopy/interferometry. These analyses may be useful in designing and optimizing HCF-based gas sensors.

## Figures and Tables

**Table 1:** Filling times measured in various systems with photonic band-gap (PBG) HCFs.

Reference	Fiber Length (m)	Approximate Diffusion Time (minutes)	Core Diameter (µm)	Sample Gas
[35]	5.1	7	12	CH_4_
[38]	0.55	4	− ^1^	H_2_S
[39]	0.74	10	14.5	CO_2_
[40]	1	20	10.5	CO_2_
[40]	20	90	10.5	CO_2_
[41]	0.3	60	10	CH_4_
[42]	0.45	80	10	CH_4_

^1^ Core diameter was not provided in the paper.

**Table 2:** Advantages and drawbacks of PBG HCFs when used for trace gas sensing.

Advantages	Drawbacks
• Commercially available with lengths beyond tens of meters	• Very long filling times
• Can be easily spliced with other silica-based fibers using standard fusion splicers	• Optical fringes usually visible in the background
• Low losses	• Expensive

**Table 3:** Summary of the performance of gas sensing with NC HCFs reported in the literature.

Ref.	Fiber Length	Diffusion Time (s)	λ (µm)	Target Molecule	Detection Technique	Detection Limit
[11]	1.35 m	5	2	CO_2_	WMS	5 ppm in 1 s
[65]	1 m	19	1.57 and 3.33	CO_2_ and CH_4_	WMS	63 ppb (CH_4_) and 153 ppm (CO_2_) in 1 s
[66,72]	85 cm	5 [72]	2.3	CO	TDLAS, WMS, PTI	0.4 ppm in 30 s [72]
[67]	3.2 m	23	4.53	N_2_O	TDLAS, WMS	5.4 ppb in 1 s
[70,71]	1.3 m	<10	3.33	CH_4_	WMS, CLaDS	500 ppb in 1 s [71]
[73]	1.2 m	not specified	3.6	N_2_O	TDLAS	35 ppm (TDLAS)
[74]	74 cm	44 ^1^	1.53	C_2_H_2_	PTI	15 ppt in 100 s ^2^
[82]	1.15 m	9	5.26	NO	TDLAS, WMS	30 ppb in 1 s

^1^ Gas freely diffused through side microchannels, not through fiber ends. ^2^ Result obtained with a 4.67-m-long HCF.
